# Natural Products as Sources of Antimalarial Drugs: Ethnobotanical and Ethnopharmacological Studies

**DOI:** 10.1155/2020/7076139

**Published:** 2020-05-09

**Authors:** Oluwole Solomon Oladeji, Abimbola Peter Oluyori, Deborah Temitope Bankole, Tokunbo Yemisi Afolabi

**Affiliations:** Natural Products Research Unit, Department of Physical Sciences, College of Pure and Applied Sciences, Landmark University, PMB 1001, Omu Aran, Kwara State, Nigeria

## Abstract

**Materials and Methods:**

For this study, relevant information was procured from the inhabitants via a structured questionnaire to procure the general knowledge of antimalarial medicinal plants. *Results and Discussion*. A total of 90 interviewees (44 men and 46 women) were involved in this survey. A total of 59 medicinal species were identified, which were dispersed in 33 families (Asteraceae (6), Apocynaceae (5), Anacardiaceae, Annonaceae, Fabaceae, Malvaceae, Meliaceae, Poaceae, and Rubiaceae (3 each), Phyllanthaceae (2)) totaling 49% of the cited species. The most cited plants are *Azadirachta indica* (42), *Mangifera indica* (38), *Carica papaya* (28), *Cymbopogon citratus* (27), *Cassia fistula* (15), *Morinda lucida* (14), *Anacardium occidentale and Vernonia amygdalina* (13 each), *Helianthus annuus* (11), *Enantia chlorantha* (10), and *Moringa oleifera* (9) A total of 105 citations were recorded for the plant parts used (leaf (46), bark (17), fruits (9), root (9), latex (11), stem (11), and inflorescence (2)) while decoction (59%), maceration (25%), infusion (9%), and exudation (7%) were the methods of preparation. Use Values (UVs) of 0.47 to 0.11 were recorded for the frequently used antimalarial plants. The Efficiency Levels (ELs) of 11 different medicinal plants stated by the respondents were *Azadirachta indica*, *Cassia fistula* and *Morinda lucida* (12), *Chromolaena odorata* (10), *Mangifera indica, Enantia chlorantha* and *Helianthus annuus* (8), *Cymbopogon citratus* (7), *Gossypium arboretum* (4), *Landolphia dulcis* (3), and *Aloe vera* (2) *Cocos nucifera*, *Curcuma longa*, *Forkia biglobosa*, and *Musa acuminate* are mentioned for the first time in the study area with little or no reported antiplasmodial activities.

**Conclusion:**

The study appraised the commonly used antimalarial plants in the study areas. Therefore, commitment to scientifically explore the bioactive compounds, antimalarial potential and toxicological profile of these plants is inevitable as they could lead to novel natural products for effective malaria therapy.

## 1. Introduction

Malaria is one of the communal diseases of man contributing to stern sociocultural, economic, and health influences in humid, middle-income nations, sub-Saharan Africa, Southeast Asia, and South America [1, 2]. It is instigated by *Plasmodium ovale*, *P. malariae*, *P. falciparum*, *P. vivax*, and *P. knowlesi* [3]. The outburst of malarial infections in Africa, the Caribbean, Asia, and South American is symptomatic of *P. falciparum*, the most lethal malaria parasite. The parasite could accumulate in the brain capillaries [4, 5]. Malarial infections in India, Central American, and East Mediterranean could be concomitant to *P. vivax* while *P. ovale* and *P. malariae* are prevalent in Papua New Guinea and sub-Saharan Africa [6].

Malaria epidemic has been enormously high in low socioeconomic empowered regions. In Africa, nearly 19 million cases of malaria infections have been reported accounting to 89% of the global cases and almost 17 million deaths have been published [7]. Also, about 450 thousand African children's deaths have been reported and one-tenth of pregnancy deaths have been concomitant to malaria infections [2, 8]. It affected the morbidity and mortality rate owing to pathogenic resistance to conventional drugs, vector control agents, and human migration [9]. Several factors have been analysed and reported to control malaria infections in Africa. These are climate suitability, dams or reservoirs, migration, and vegetation. According to the report published by The World malaria in 2018, malarial cases have tremendously reduced in relation to the report of 2010. Despite this, between 2015 and 2017, no significant progress was achieved in curbing malaria cases [10]. This trend could be indicative of the widely spread of drug-resistant malaria and the intricacy of parasites' life cycle [11].

Quinoline (QN) derivatives are undoubtedly the commonest antimalarial drugs in Africa. Examples of quinoline antimalarial drugs are quinine, amodiaquine, piperaquine, primaquine, pyronaridine, ferroguine, isoquine, amopyroquine, tertbutylisoquine, mefloquine, tafenoquine, and chloroquine. 4-Aminoquinoline is the most accessible antimalarial pharmacophore used in the last century. In recent times, QN derivatives have been integral component of Artemisinin-based Combination Therapy (ACT) [12]. The discovery of ACT could be considered as the most noteworthy achievement of ethnopharmacological research in the 20th century [13–15], enthused by the use of *Artemisia annua* L. (Asteraceae). The drug was found effective against all the malarial parasites and led to regulations against quinine-based drugs in Africa. However, despite the predominant achievements of ACT, concerns about the future efficacy of artemisinin have recently been on the rise due to the building-up of resistance by the parasite [7]. This event instigates the unrelenting search for promising antimalarial drugs that are cost-effective, handy, acceptable, and scientifically proven.

Human has used medicinal plants for malaria, cholera, yellow fever, and diabetes treatment [16]. In most African countries, medicinal herbs are viewed as alternative therapies. Medicinal plants have effectively helped in primary health care for the therapy of acute and chronic diseases [17, 18]. They have contributed to the discovery of novel therapeutic agents via isolation, identification, and characterization of secondary metabolites [19]. Secondary metabolites such as flavonoids, stilbenes, coumarins, lignin, tannins, terpenoids, and steroids have been reported as antimalarial compounds [20].

Tropical plants are identified to contain high proportions of natural chemical compounds and a greater diversity than plants from any other biome. Thus, they are potential sources of new medicines [21]. The increased number of drug-resistant strains makes the development of novel antimalarial urgent. The high cost of malaria treatment has left the poor masses of Nigeria heavily reliant on traditional practitioners and medicinal plants for the treatment of the disease. It seems logical to encourage studies on plants from these regions, especially since the major proportions of malaria attributable deaths occur in sub-Saharan African regions. Although several compounds had achieved success at treating malaria diseases, the emerging threats of drug resistance by some plasmodium species call for the development of new molecules with novel bioactive features. The study explores the ethnobotanical and ethnopharmacological appraisal of antimalarial plants used by people of Omu Aran, Ogbomoso, Ado Ekiti, and Sagamu communities in Nigeria. Hence, the search for novel natural antimalarial molecules in selected plant sources via ethnobotanical and ethnopharmacological investigation is clearly justified.

## 2. Methodology

### 2.1. Geographical Description of the Study Area

**Figure 1:**
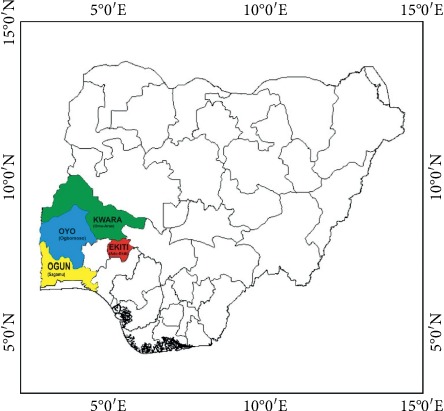
Map of Nigeria showing the study area.

The study area comprises four states, namely, Kwara (Omu Aran), Oyo (Ogbomoso), Ekiti (Ado Ekiti), and Ogun (Sagamu) in Nigeria located on 8°08′N (5°06′E), 8°08′N (4°15′E), 7°37′16″N (5°13′17″E), and 6°50′N (3°39′E), respectively. The study areas are located in two important geopolitical zones, that is, Omu Aran (North central), Ogbomoso, Sagamu, and Ado Ekiti (Southwest) of Nigeria (Figure 1) The inhabitants are majorly from the Yoruba ethnic group. The study area falls into the category of state with most prevalence of malaria in Nigeria according to MIS report.

### 2.2. Typical Vegetation of the Study Area

Ado Ekiti and Sagamu fall in the rain forest region, characterized by temperature of 21° to 28°C, high humidity, and two distinct seasons, rainy season from April to October and dry season from November to March with mean annual rainfall of 1320 mm. Ogbomoso and Omu Aran fall in the savanna region, characterized by temperature of 21° to 33°C with heavy rainfall between April and October. The humidity is high (51.1%) with mean annual rainfall of 1885 mm. The study sites are opulently rich in evergreen floras and this promotes the use of local herbs for diseases prevention and cure.

### 2.3. Selection of the Informants

For this study, relevant information and data were procured from selected people in the study area via interview using structured questionnaire to procure relevant knowledge of antimalarial plants used in the vicinity. The questions were structured in a simple way and interpreted to selected respondents selected by nomination method after verbal authorization and approval by the chiefs in the study areas. In a particular study area, the leaders suggested prominent people with vast experience in herbal medicines or practitioners of herbal medicines. For reliability and reproducibility, respondents that accepted to be interviewed were briefed on the significance and objectives of the study. A disclaimer was presented to the interviewees that the views, ideas, and opinions expressed belong solely to the interviewers, and not necessarily to any committee or individual. While conducting the research, researchers were honest but not too detailed in briefing the respondents what he or she needed to do. Conducting the survey involved series of activities. These include establishing cordial relationship with respondents, selecting easy ways of interacting, observation, and recording the findings.

Respondents selected must meet the following criteria: (1) they should be indigenous people of Yoruba; (2) they are sound and knowledgeable in phytotherapy; (3) they are accessible to medicinal plants; (4) they must have used herbs for treating malaria; (5) they are approachable and organized.

### 2.4. Structured Questionnaire

The structured questionnaire was designed according to the technique of Olorunnisola et al. [22] and Sarquis et al. [23] with slight modification. The moderated questionnaire entails information on respondent biodata, commonly used antimalarial medicinal plants, plant parts frequently used, the most effective herbs from the respondents list, mode of preparation, and common side effects of antimalarial plants.

### 2.5. Data Collection

The study was piloted for 6 months, from March to August 2019. The mode of data collection was through one-on-one interviews, public discussion, and observation. The interviews were conducted mostly in Yoruba (native) language. The respondents gave the native names of plants and showed the interviewers the available plant samples. Information on the questionnaires was supplied on the spot of interview, and several observations and discussions were conducted prior to completing and cross-checking of the information provided.

### 2.6. Data Analysis

The antimalarial medicinal plants itemized by the respondents were structured according to the scientific, common, and local names, family, plant part used, and mode of preparation. The malarial diseases' symptoms and probable health effects or body reactions were reported. Data were statistically analysed in percentages using Graphpad Prism software (version 6.0) The comparative significance of a plant species for its ethnopharmacological activity was evaluated with the Index of Use Value (UV) and efficiency level (EL).

#### 2.6.1. Use Value (UV)

It is a quantifiable catalogue that denotes the therapeutic importance of each medicinal plant species. It is calculated by UV = Σ*Ui*/*n*, where Ui is the total number of times plant species is cited and *n* is the total number of respondents interviewed. UV element helps evaluate plant species frequently mentioned for antimalaria. A high UV denotes plant mentioned mostly by respondents and low for sparingly mentioned [23].

#### 2.6.2. Efficiency Level (EL)

It is a qualitative index that signifies the efficacy of a single plant species from the list of plants given as a response by the interviewees. EL is calculated by CL = *Ui*, where *Ui* is the total number of times a particular plant species is mentioned as the most effective from the list of plant species level. EL indicates plant species showing the most effective therapeutic potentials. A high EL denotes the most efficacious plant.

## 3. Results and Discussion

### 3.1. The Demographic Details of the Informants

**Figure 2:**
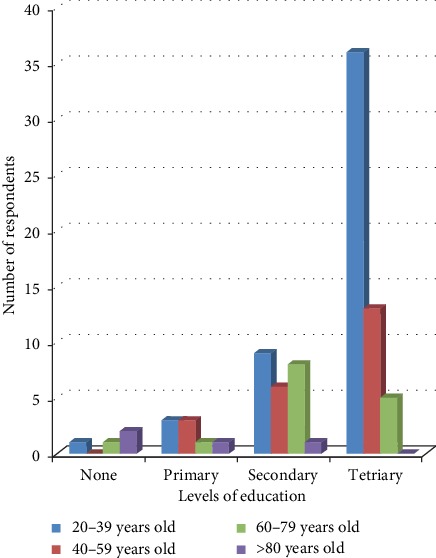
Age distribution and level of education of the respondents.

A total of 90 interviewees (44 men and 46 women) were involved in this ethnobotanical and ethnopharmacological survey. Demographic details of the interviewees are listed in Table 1. The respective age distribution and the level of education of the respondents are shown in Figure 2.

### 3.2. The Effectiveness of the Medicinal Plants

In this study, 57 respondents (63%) strongly agreed and 26 respondents (29%) agreed that malaria is curable using medicinal herbs while 7 respondents (8%) were neutral. This denotes the local belief in phytotherapy of malaria. The study site has rich vegetation diversity ranging from creeping plant to shrubs and trees. A large number of these plants are used by the inhabitants in malaria therapy due to persistent spread of malaria in these regions.

### 3.3. Indigenous Notion of the Study Area on Malaria

The common symptoms of malaria and side effects of antimalarial plants according to the native knowledge of Ado Ekiti, Ogbomoso, Omu Aran, and Sagamu people are detailed in Table 2. Yoruba people identify malaria as “iba” and presumed malaria as a common and seasonal disease. From one-on-one interview and observations, malaria prevalence is significantly high during the rainy season in the study areas. According to the respondents, malaria is caused by long-time exposure to rain, cold, hot sun, stress, and mosquito. They believed that these could disrupt the temperature balance in the body.

**Figure 3:**
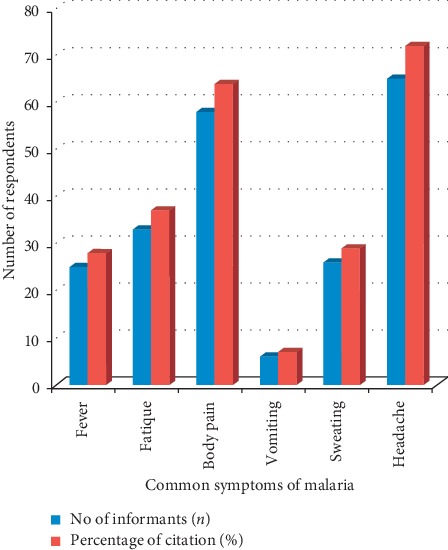
Common symptoms of malaria.

Likewise, the respondents were screened to procure knowledge of malaria via the common symptoms they experienced (Figure 3) Fever, body pain, fatigue, and headache are the common symptoms in the study area and are related to temperature balance of the body system. The local people believed that it could be caused by excessive heat and long-time exposure to cold environment which forces the body to produce excessive heat. Moreover, they explained that fever could lead to other symptoms such as headache, fatigue, body pain, and sweating. According to the respondents, probable ways of preventing malaria include reduction in exposure to rain (cold areas) or sun (hot areas), avoidance of mosquito bites, reduction in workload (stress), constant use of antimalarial herbal drugs, and burning of aromatic antimalarial plants which could pose threats to mosquitoes.

**Figure 4:**
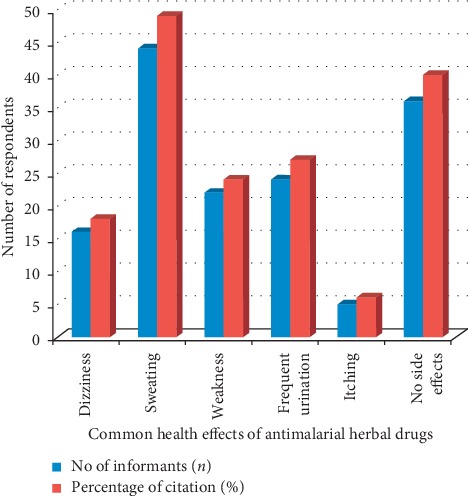
Health effects of antimalarial herbal drugs.

Medicinal plants are universally reported to produce uncharacteristic effects ranging from simple to intricate. The respondents were screened to procure information on common health effects associated with antimalarial herbal drugs. Several reports were obtained, grouped as dizziness, sweating, weakness, frequent urination, itching, and no side effects (Figure 4) However, 15 (70%) respondents cited other effects produced by medicinal plants on their body systems. The respondents believed that these effects are related to the nature of medicinal plants combined, quantity of herbs taken, period when herb is used, temperature of herbal drugs (warm or cold), season drugs are taken, severity of malaria, and body capacity.

### 3.4. Assortment of Antimalarial Therapeutic Plants

A total of 59 medicinal plants were cited which belong to 33 families. These are Asteraceae (6), Apocynaceae (5), Anacardiaceae, Annonaceae, Fabaceae, Malvaceae, Meliaceae, Poaceae and Rubiaceae (3 each), Phyllanthaceae (2) totaling 48.83% of the sampled species while Asteraceae, Arecaceae, Asphodelaceae, Boraginaceae, Bromeliaceae, Caricaceae, Crassulaceae, Lamiaceae, Lythraceae, Menispermaceae, Moringaceae, Musaceae, Rutaceae, Sapindaceae, Myrtaceae, Solanaceae, Zingiberaceae, Solanaceae, Meliaceae, Theaceae, Labiatae, Hymenocardiacae, and Zingiberaceae accounted for 22.5% of families mentioned once (Table 3) The most cited plants include *Azadirachta indica* (42), *Mangifera indica* (38), *Carica papaya* (28), *Cymbopogon citratus* (27), *Cassia fistula* (15), *Morinda lucida* (14), *Anacardium occidentale and Vernonia amagdalina* (13 each), *Helianthus annuus* (11), *Enantia chlorantha* (10), *Moringa oleifera* (9), *Chromolaena odorata*, and *Psidium guajava* (7 each) The efficacy of a plant species is evidenced in its number of citations, thus, becoming spotlight in pharmacological research leading to the discovery of novel antimalarial drugs. However, we cannot rule out the possibility of cultural factors unrelated to efficacy as having impacted the citation rate.

### 3.5. Used Medicinal Plant Parts

**Figure 5:**
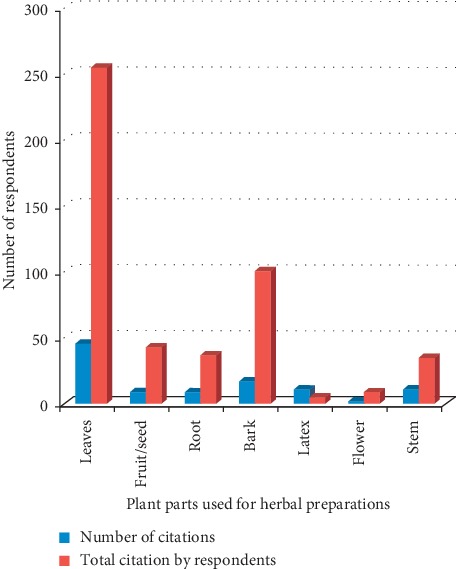
Plant parts used for herbal preparations.

The commonest used parts cited are leaf (46), bark (17), fruits (9), root (9), latex (11), stem (11), and inflorescence (2) (Figure 5) Many antimalarial herbal drugs are commonly prepared from a single plant part, although they could be prepared from the assortment of two or more plant parts. In this survey, leaf and bark were the most cited plant parts contributing to 255 and 101 of the 480 plant parts cited by the respondents. Leaves are the most commonly used plant parts in Nigeria [24, 25]. This could be due to the simplicity of the collection, site of synthesizing majority of plant secondary metabolites, and diverse bioactive compounds appraised by preliminary phytochemical investigations of leaves [26–28]. Systematic harvest of leaves has little or no influence on plants survival. This explains the frequent utilization of leaves in herbal recipes [29, 30].

### 3.6. Forms of Herbal Drugs' Preparations for Malaria Therapy

**Figure 6:**
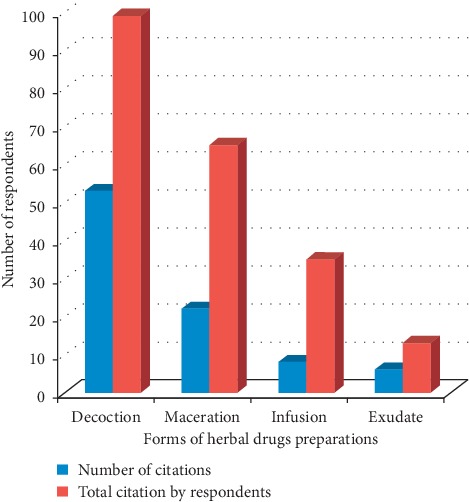
Forms of herbal drugs preparations for malaria therapy.

The common herbal drugs' preparations according to the study were categorized as decoction, maceration, infusion, and exudation (Table 3) The most cited methods of preparation are decoction (59%), maceration (25%), infusion (9%), and exudation (7%) (Figure 6) Decoction was cited 99 times; maceration, 65 times; infusion, 35 times; and exudation, 13 times. Decoction is commonly used in herbal recipes because recipe could be stored, could have long-life span, could be taken orally, and could be used as bath. Due to heat treatment, recipe is safe to administer and more metabolites are believed to be extracted. Maceration is also common among the Yorubas. It involves permeation of the plant materials (mostly bark and root) in aqueous (water) or organic (alcohol) solvents.

### 3.7. Assessment of the Different Indexes

In this study, UVs within 0.47 and 0.11 is appraised as frequently used antimalarial plants by the Yorubas: *Azadirachta indica* (0.47), *Mangifera indica* (0.42), *Carica papaya* (0.31), *Cymbopogon citratus* (0.3), *Cassia fistula* (0.17), *Morinda lucida* (0.16), *Anacardium occidentale* (0.14), *Vernonia amagdalina* (0.14), *Helianthus annuus* (0.12), and *Enantia chlorantha* (0.11) (Table 3) The most significant plant species are those with high UV and should be compiled for preservation.

The EL appraised the efficacy of a particular plant from the catalogue given by the interviewees. In this study, 11 different medicinal plants were mentioned by the respondents as most efficacious from array of medicinal plants listed. 26 respondents cited *A. indica* and *C. fistula* while *M. lucida* was cited by 12 respondents; *C. odorata*, 10 respondents; *M. indica, E. chlorantha,* and *H. annuus,* 8 respondents each; *C. citratus* (7 respondents); *G. arboretum*, 4 respondents; *L. dulcis*, 3 respondents; and *A. vera*, 2 respondents.

The *in vitro* and *in vivo* antiplasmodial potency of medicinal plants has been appraised against *P. falciparum*, *P. berghei*, and *P. yoelii.* Some of the plants with exceptional antiplasmodial activities are *P. guajava* [31], *N. latifolia* [32], *C. citratus* [33, 34], *U. chamae* [35], *E. chlorantha* [36, 37], *O. gratissimum* [38], *A. leiocarpus* [39], *P. amarus* [40], *A. indica* [41, 42], *C. odorata* [43, 44], *M. lucida* [45, 46], *V. amygladina* [47], *A. boonei* 44, 48], *A. senegalensis* [48], *A. occidentale* [49, 50], *B. ferruginea* [51], *G. arboretum* [48], *M. oleifera* [39], and *S. jollyanum* [44].

About four plant species are mentioned for the first time as antimalarial medicinal plant. These plants have a low UV indicating that there is little awareness on these plants in the region. The plants are *Cocos nucifera* (0.01), *Curcuma longa* (0.01), *Forkia biglobosa* (0.01), and *Musa acuminate* (0.01).

### 3.8. Antimalarial Assays of Medicinal Plants

Herbal plants are essential part of biodiversity which have proven to ease and remediate several diseases and infections. In tropical African countries, herbal medicine has been an undisputable therapeutic medium as alternative to conventional medicine [52]. In view of this, therapeutic potentials of medicinal plants are appraised against numerous diseases such as malaria, diabetes, cancer, ulcer, hypertension, and viral infections [53]. Generally, pharmacological activities of medicinal herbs could be linked to the existence of secondary metabolites like cardiac glycosides, saponins, tannins, flavonoids, terpenoids, and alkaloids [18]. Several plants have been explored for their antimalarial potency with curative basis exploited from ethnopharmacological beliefs (Table 4) [76].

*Cymbopogon citratus*. Lemongrass (Poaceae) is a perennial grass, evenly distributed in the tropic region, South and Central America, and has an outstanding profile in the folk medicine [17]. The antimalarial potential of aqueous leaf extracts of *C. citratus* assessed on twenty-five Swiss albino mice demonstrated significant prophylactic and chemotherapeutic potency against mice infected with 0.2 ml O^+^ human parasitized blood of *P. falciparum* after 72 h. Significant inhibition was observed in parasitaemia level of blood of infected mice [55]. A larvicidal test of geranial, an essential oil in *C. citratus*, was evaluated against *Anopheles funestus* (mature larvae) and *P. falciparum* according to the WHO standard procedure. Prominent activities were recorded at LD_50_ (35.5 ppm and 34.6 ppm) after 6 h. Geranial also displayed significant antiplasmodial activity with IC_50_ (4.2 ± 0.5l g/mL) when assessed by the radioisotopic method. Geranial could serve as effective natural biocides for combating the larvae of malaria vectors [61]. The antiplasmodial activity of aqueous leaf and root extracts of *C. citratus* (200, 400, and 800 mg/kg) and chloroquine (5 mg/kg) was examined against *P. berghei* in mice using 4-day suppressive test model at *P* < 0.05. A dose-dependent suppressive pattern was observed with chloroquine and 800 mg/kg (aqueous root extract) [67]. *C. citratus* plant displayed significant antimalarial activity than herbal concoction or chloroquine (3200 mg/kg) (control) when used as a prophylactic treatment against CBA/Ca mice with patent *P. berghei* ANKA or *P. chabaudi* AS at doses of 1600 and 3200 mg/kg. In addition, the synergetic activity of chloroquine and *C. citratus* plant exhibited high activity than chloroquine alone against *P. berghei*. The antimalarial activity of entire *C. citratus* plant aids inevitable efforts to developing whole plant remedies for the treatment of malaria [65].

*Morinda lucida.* The antimalarial investigation of partly purified cysteine-stabilised peptide extracts of *M. lucida* leaf was assessed *in vitro* against *P. falciparum* W2 and its activities on certain liver and erythrocyte antioxidant parameters in *P. berghei* NK65-infected mice. Low activities were observed in *P. falciparum* W2 (IC50: >50 *μ*g/ml); however, *in vivo* activity against *P. berghei* led to 51.52% reduction in parasitaemia on 96 h after inoculation and considerably decreased (*P* < 0.05) malondialdehyde concentrations in the liver and erythrocyte at high doses in contrast to untreated controls [60]. N-Hexane and chloroform fractions of *M. lucida* leaf extract conducted using standard techniques showed significant activities at 0.6 mg/ml [64]. The antimalarial activities of *M. lucida* investigated in *P. berghei*-infected mice exhibited dose-dependent chemosuppression of 39.8–90.5 which show pronounced activities than quinine [58].

*Enantia chlorantha. Enantia chlorantha* Oliver (or *Annickia chlorantha*) belongs to Annonaceae family, so-called Awopa, Osu pupa or Dokita igbo, Eru meru, Kakerim, and Erenba-vbogo in Nigeria. It is dense and widely distributed in Nigeria, Angola, Gabon, Cameroon, and Congo [77]. Oral administration of aqueous extract of *E. chlorantha* inhibited *Plasmodium yoelii* in mice at 0.2 to 150 mg/ml while ethanolic extract inhibited the parasite at dose of 0.05 to 0.5 mg/g. The ethanolic and aqueous extracts have ED_50_ values of 0·34 mg·g^−1^and 6·9 mg·g^−1^ which are schizonticidal in the mode of action. The activities could be linked to the presence of saponins, tannins, simple sugars, and alkaloids [78]. Synergic reactions of *E. chlorantha* with *N. latifolia* and *A. altilis* were reported to display significant antimalarial and prophylactic activities. This justifies the ethnomedical practice of combination of antimalarial herbal therapies in combating acute or chronic malaria [63].

*Aloe vera*. The methanolic extracts of *Aloe vera* were assessed *in vivo* for its antiplasmodial potency against *P. falciparum* strain with 50% inhibition of 32 to 77 *μ*g/ml. The anthrone C-glucoside homonataloin isolated inhibited the strains with activity of 13.46 ± 1.36 *μ*g/ml (IC_50_); similarly, homonataloin displayed activities of 107.20 ± 4.14 *μ*g/ml (IC_50_) [79]. C-glycosylated anthrones, that is, nataloin and 7-hydroxyaloin, two isolated compounds in *Aloe pulcherrima*, displayed significant dose-independent activities on plasmodia strain using 4-day suppressive test. Pronounced activity of 56.2% was observed at 200 mg/kg/day in 48 h, which support the ethnomedical claims of the plant [80].

*Carica papaya.* The antimalarial property of *Carica papaya* leaf extracts was screened against *P. falciparum* 3D7 and Dd2 strains using bioassay-guided fractions and dichloromethane extract. The petroleum ether and chloroform fractions of *C. papaya* fruit and root assessed *in vivo* for antimalarial activity against early *P. berghei* infection in mice displayed pronounced chemosuppressive effect at *P* < 0.001. Significant activities were observed in petroleum ether fractions (61.78%) compared to 48.11% of chloroform fraction [71]. The synergistic effects displayed by the administration of *C. papaya* and *V. amygdalina* in ameliorating plasmodium infection in mice showed significant result at *P* < 0.05. The oral administration significantly surged the RBC and PCV renaissance when compared to the disease control. This underlined the importance of plants in conventional therapy of malaria infection [68]. Ethanolic leaf extract of *C. papaya* was appraised on chloroquine-sensitive and chloroquine-resistant strains of *P. falciparum.* The extracts significantly inhibited the activities of both plasmodium strains with IC_50_ = 40.75%, 36.54%, 25.30%, and 18.0% for chloroquine-sensitive and IC_50_ = 50.23%, 32.50%, 21.45%, and 23.12% for chloroquine-resistance plasmodium strains [81].

*Azadirachta indica. Azadirachta indica* extract is appraised to contain bioactive compounds which dictate its potencies against *P. vivax* and *P. falcifarum* [82]. Ethanolic leaf extracts assessed *in vivo* absolutely inhibited *P. berghei* growth, at azadirachtin dosage of 50 mg/kg mouse body weight [83]. The *in vivo* antiplasmodial potency of aqueous and ethanolic leaf extracts was examined in *P. berghei*-infected BALB/c mice at dosage of 50 to 200 mg/kg/day. Both extracts exhibited significant antiplasmodial potency in a dose-dependent technique which could be due to the active antiplasmodial compounds screened [56].

## 4. Conclusion and Future Prospects

Malaria is a universal civic health peril, and recent drug resistance of the parasite is a persistent concern. This study shows that a highly diverse set of native medicinal herbs is currently used for the management of malaria in Nigeria. Based on the results, there is substantial indication that the traditional use of antimalarial medicinal plants by Yoruba ethnics (studied areas) is driven by important therapeutic agents, which could be elucidated structurally and further established by *in vitro* or *in vivo* investigations. In recent times, the growing interest in phytoremediation of malaria led to the isolation and characterization of bioactive compounds in medicinal plants (Table 5) The isolation, characterization, and quantification of these compounds were appraised via chromatographic and spectrophotometric methods. Likewise, different assays such as susceptibility microassay technique [95], four day suppressive test [96], 96-well microtiter plate format SYBR green florescence assay [97], and LDH method [98] are used to appraise the antiplasmodial potential of plant extracts (Table 4).

Several modes of preparation, usage factors, health risks, and countermeasures on the use of antimalarial herbal drugs should be systematically examined through advanced scientific approaches. This will aid in the identification and authentication of therapeutic potency of antimalarial compounds isolated from medicinal herbs, thereby promoting its global relevance as efficacious and safe antimalarial plants in primary health care. Individuals, societies, sociogroups, and governmental and nongovernmental organizations should devise plans which could assist in the conservation of these medicinal plants in order to prevent their extermination and exploitation of indigenous populations, as well as considerations for cultural disruptions should one or more of these plant species become a valuable resource. In the meantime, the outcomes of this study serve as a platform of appraisal for indigenous claims of medicinal plants as effective antimalarial drugs in Nigeria and the world as a whole.

## Figures and Tables

**Table 1:** Demographic details of the informants (*N* = 90).

Biodata	Group of informants	No of informants, *n* (%)
*Age*	20–39 years old	49 (54.44)
	40–59 years old	22 (24.44)
	60–79 years old	15 (16.67)
	>80 years old	04 (4.44)
*Sex*	Male	44 (48.88)
	Female	46 (51.11)
*Education*	Illiterate (none)	16 (17.78)
	Primary level	07 (7.78)
	Secondary level	23 (25.56)
	Tertiary level	44 (48.89)
*Location*	Urban	54 (60.00)
	Rural	36 (40.00)

**Table 2:** The common symptoms of malaria and the health effects of antimalarial herbal drugs.

Common symptoms of malaria	No of informants, *n* (%)	Health effects of antimalarial herbal drugs	No of informants, *n* (%)
Fever	25 (28)	Dizziness	16 (18)
Fatigue	33 (37)	Sweating	44 (49)
Body pain	58 (64)	Weakness	22 (6)
Vomiting	6 (7)	Frequent urination	24 (5)
Sweating	26 (29)	Itching	5 (6)
Headache	65 (72)	No side effects	36 (40)

**Table 3:** The medicinal plants used as antimalarial in Nigeria (Omu Aran, Ogbomoso, Ado Ekiti, and Sagamu).

Botanical name	Local name(s)	Family name	Parts used	Common method of preparation
(1) *Acanthospermum hispidum* (starburr, goat head)	Dagunro	Asteraceae	Stem, leaves	Decoction, maceration
(2) *Ageratum conyzoides* (billygoat-weed, goatweed, chickweed, whiteweed)	Imi-esu	Asteraceae	Leaves	Decoction
(3) *Anogeissus schimperi*	Ayin	Combretaceae	Leaves, bark	Decoction, maceration
(4) *Aloe vera* (Aloe)	Ahon erin	Asphodelaceae	Leaves	Exudate
(5) *Alstonia boonei* (cheese wood, stool wood)	Ahun	Apocynaceae	Bark, root	Decoction, infusion
(6) *Anacardium occidentale* (cashew)	Kasu	Anacardiaceae	Stem, leaves, bark	Decoction, infusion, maceration
(7) *Ananas comosus* (pineapple)	Eso alade, ope oyinbo	Bromeliaceae	Unripe fruit	Exudate, decoction
(8) *Annona senegalensis* (African custard apple, wild soursop)	Arere	Annonaceae	Root	Infusion, maceration
(9) *Azadirachta indica* (neem, Indian lilac, nimtree)	Dogoyaro, eka ebo	Meliaceae	Bark, leaves, root,	Decoction, infusion, maceration,
(10) *Bridelia exaltata* (scrub ironbark, brush ironbark)	Ira, iran oda, ira eju	Phyllanthaceae	Bark	Decoction, maceration
(11) *Bryophyllum pinnatum* (cathedral bells, miracle leaf, life plant)	Abamoda	Crassulaceae	Leaves	Decoction
(12) *Calotropis procera* (sodom apple, rubber bush)	Bomu-bomu	Apocynaceae	Leaves, fruit	Decoction, exudate
(13) *Camellia sinensis* (tea bush)	Werepe	Theaceae	Leaves	Decoction
(14) *Capsicum frutescens* (chili pepper)	Ata-ijosi, ata-wewe	Solanaceae	Seed/fruit	Maceration, exudate
(15) *Carica papaya* (pawpaw)	Ibepe	Caricaceae	Fruit, leaves, root	Infusion, maceration
(16) *Cassia fistula* (golden shower, Indian laburnum)	Igi kasia	Fabaceae	Stem, leaves, bark	Decoction, infusion
(17) *Ceiba pentandra* (kapok tree)	Iroko	Malvaceae	Leaves	Decoction
(18) *Chromolaena odorata* (siam weed, devil weed, Christmas bush)	Ewe akintola, awolowo,	Asteraceae	Leaves, stem	Decoction
(19) *Citrus aurantifolia* (lime, key lime, west Indian lime, bartenders lime)	Orombo	Rutaceae	Leaves, fruit	Decoction, exudates
(20) *Citrus aurantium* (sour orange, bigarade orange, bitter orange)	Osan jagan	Rutaceae	Leaves, fruit	Decoction, exudates
(21) *Citrus limon* (lemon)	Osan wewe, ilamuna	Rutaceae	Stem, root, leaves, fruit	Decoction,
(22) *Citrus paradise* (grape)	Ajara	Rutaceae	Fruit	Exudates
(23) *Cocos nucifera* (coconut)	Agbon	Arecaceae	Fruit shell	Decoction, infusion
(24) *Curcuma longa* (turmeric)	Ata-ile pupa	Zingiberaceae	Fruit	Decoction, maceration
(25) *Cymbopogon citratus* (lemongrass, Malabar grass)	Oka oyinbo, ewe tea, koko oba	Poaceae	Leaves,	Decoction
(26) *Enantia chlorantha* (African yellow wood)	Awopa, dokita igbo, osu pupa	Annonaceae	Leaves, bark	Decoction, maceration
(27) *Parkia biglobosa* (African locust bean, eggplant)	Igi iru, sumbala	Fabaceae	Leaves, bark	Decoction, maceration
(28) *Gardenia ternifolia*	Oruwon, Gangan	Rubiaceae	Leaves	Decoction
(29) *Gongronema latifolium* (bush buck)	Arokeke	Apocynaceae	Leaves, bark	Decoction, maceration
(30) *Gossypium arboretum* (cotton plant)	Owu	Malvaceae	Leaves	Decoction
(31) *Helianthus annuus* (sunflower)	Fufulele, June 12, agunmoniye	Asteraceae	Leaves	Decoction,
(32) *Heliotropium indicum* (Indian heliotrope, turnsole, English combs comb)	Agogo igun, ogbe akuko, akuko omade	Boraginaceae	Stems, leaves, root, bark	Decoction, maceration, infusion
(33) *Hibiscus sabdariffa* (Roselle, carcade)	Zobo	Malvaceae	Flower	Decoction, infusion
(34) *Hoslunda opposite*	Efirin	Labiatae	Leaves	Decoction
(35) *Hymenocardia acida*	Aboopa, orupa	Hymenocardiacae	Leaves	Decoction
(36) *Khaya grandifoliola* (African mahogany)	Oganwo	Meliaceae	Bark	Maceration
(37) *Lactuca canadensis* (Canada lettuce, tall lettuce)	Yanrin	Asteraceae	Leaves	Decoction
(38) *Landolphia dulcis*	Ibobo, ibo	Apocynaceae	Leaves	Decoction
(39) *Lawsonia inermis* (Henna, Egyptian privet, cypress shrub)	Laali	Lythraceae	Leaves	Decoction
(40) *Lecaniodiscus cupanioide*	Akika	Sapindaceae	Leaves	Decoction
(41) *Mangifera indica* (mango)	Mangoro, oro	Anacardiaceae	Bark, stem, leaves	Decoction, maceration, infusion
(42) *Morinda lucida* (brimstone-tree)	Oruwo	Rubiaceae	Leaves	Decoction
(43) *Moringa oleifera* (moringa, drumstick)	Ewe ile, igbale igi iyanu	Moringaceae	Leaves, bark	Decoction, maceration
(44) *Musa acuminate* (banana)	Ogede	Musaceae	Leaves	Decoction
(45) *Nauclea latifolia* (African peach)	Egbo igbesi	Rubiaceae	Leaves, root bark	Decoction
(46) *Ocimum gratissimum* (clove basil, scent plant, African basil)	Efirin, aramogbo	Lamiaceae	Leaves, stem	Decoction
(47) *Panicum miliaceum* (proso millet, hog millet)	Poporo oka, oka baba	Poaceae	Stem	Decoction, maceration
(48) *Parquetina nigrescens*	Igi ogbo	Apocynaceae	Leaves	Decoction
(49) *Pennisetum purpureum* (elephant grass, napier grass, Uganda grass)	Eèsún, eèsún funfun	Poaceae	Leaves	Decoction
(50) *Phyllanthus amarus*	Eyin olobe	Phyllanthaceae	Leaves	Decoction
(51) *Senna alata* (candle bush, candletree)	Asunwon oyinbo	Fabaceae	Leaves, flower, fruit	Decoction, maceration
(52) *Sorghum bicolor* (durra, great millet, jowari)	Poroporo okababa	Poaceae	Stem	Decoction
(53) *Sphenocentrum jollyanum*	Aduro koko, akerejupon	Menispermaceae	Root	Decoction, maceration
(54) *Spondias mombin* (yellow mombin, hog plum)	Okika, akika, iyeye	Anacardiaceae	Leaves	Decoction
(55) *Swietenia mahagoni* (mahogany)		Meliaceae	Bark	Decoction, maceration
(56) *Tridax procumbens* (coatbuttons, tridax daisy)	Igbalode, muwagun	Asteraceae	Leaves	Decoction
(57) *Uvaria chamae* (finger root, bush banana)	Eru, eruju, akisan, oko aja	Annonaceae	Stem, leaves, bark	Decoction, maceration
(58) *Vernonia amygdalina* (bitter leaf)	Onugbo, ewuro	Asteraceae	Leaves, root,	Decoction, maceration
(59) *Swietenia mahagoni* (mahogany)		Meliaceae	Bark	Decoction, maceration

**Table 4:** The antimalarial activity of selected medicinal plants.

S/n	Plant name	Plant part used	Country	Plasmodium species treated	Solvent used for extraction	Model	Control	Antiplasmodial activity	Reference
1	*Icacina senegalensis*	Leaf	Nigeria	*P. berghei*	Methanol	Swiss albino mice	Chloroquine	A dose-dependent chemosuppression of the parasites was observed at different dose levels of the extract tested with a considerable mean survival time	[54]
2	*Cymbopogon citratus*	Leaf	Nigeria	*P. falciparum*	Aqueous	Swiss albino rats	Chloroquine	Significant decrease of parasitaemia levels was observed in 120 mg/kg body weight treated group	[55]
3	*Azadirachta indica*	Leaf	Ghana	*P. berghei*	Aqueous and ethanol	BALB/c mice	Distilled water (negative), artemether (positive)	Chemosuppression of 69.65, 75.76, 78.32% (ethanol) and 64.42, 70.23, 77.41% (aqueous); artemether (86.77%)	[56]
4	*A. djalonensis, A. indica, C. cajan, C. cujete, L. inermis, L. alata, M. preussii, N. latifolia, O. subscorpioidea*, and *T. glaucescens*	Stem bark, leaf, and root	Nigeria	*P. berghei*	Ethanol and aqueous	Swiss albino mice	Distilled water (negative) and chloroquine (positive)	Optimum activity was recorded on day 4. The activity was highest with water extract of the recipe at 500 mg/kg	[57]
5	*Morinda lucida, Alstonia boonei, Curcuma longa*	Leaf	Nigeria	*P. berghei*	Ethanol	Swiss albino mice	Sulphadoxine-pyrimethamine (S-P), and quinine	Chemosuppression of 39.8–90.5, 0.2–74.8, and 34.6–78.4% observed in MLE, ABE, and CLE	[58]
6	*Azadirachta indica*	Leaf	Indonesia	*P. falciparum*	Ethanol			The extract inhibited *P. falciparum* on mature schizont stage with IC_50_ of 3.86 *μ*g/ml after 32 h incubation	[59]
7	*Morinda lucida*	Leaf	Nigeria	*P. berghei*	Dichloromethane-methanol	Adult Swiss albino mice	Chloroquine	PPCPE was active against *P. berghei* NK65 *in vivo*, with 51.52% reduction in parasitaemia on day 4 after inoculation	[60]
8	*Ocimum basilicum, Ocimum canum, and Cymbopogon citratus*	Leaf	Cameroon	*P. falciparum* and mature-stage larvae of *Anopheles funestus*		Human red blood cells in RPMI 1640 medium	Giemsa-stained blood smear	IC50 = 4.2 ± 0.5 l g/mL (*C. citratus*), 20.6 ± 3.4 lg/mL (*O. canum*) and 21 ± 4.6 lg/mL (*O. basilicum*)	[61]
9	*Azadirachta indica*	Leaf	Saudi Arabia	*P. berghei*	Ethanol	Swiss albino mice	Chloroquine and artemether	Alcoholic extracts displayed no activity, ethanol extracts of neem displayed increased parasitaemia gradually from day 0 (5%, 5.1%, and 7.2%) to day 4, with mean parasitaemia of 53%	[62]
10	*Nauclea latifolia, Artocarpus altilis*, *Murraya koenigii,* and *Enantia chlorantha*	Stem bark, root, leaf	Nigeria	*P. berghei*	Ethanol	Berghei-infected mice	Pyrimethamine and chloroquine	Prophylactic and curative ED_50_ of 189.4 and 174.5 mg/kg for *N. latifolia* and chemosuppressive ED_50_ of 227.2 mg/kg for *A. altilis*	[63]
11	*Morinda lucida, Artemisia annua*	Leaf, stem bark	Nigeria	*P. falciparum*	Ethanol		Chloroquine	MIC for chloroquine is 0.6 *μ*g/ml, *M. lucida* is 0.6 mg/ml, and *A. Boonei is* 0.2 mg/ml	[64]
12	*Cymbopogon citratus*	Whole plant	Nigeria	*P. chabaudi* AS or *P. berghei* ANKA		CBA/Ca male mice	Chloroquine	As a prophylactic treatment, the whole plant exhibited higher antimalarial activity than either the herbal infusion or chloroquine	[65]
13	*Calotropis gigantea*	Leaf, stem, and flower	India	*P. falciparum* (3D7 strain) and *P. berghei* (ANKA)	Methanol, ethyl acetate, and chloroform	Infected BALB/c albino mice	Chloroquine	Methanolic extract of leaves showed highest antimalarial activity with IC50 value of 12.17 *μ*g/ml	[66]
14	*Cymbopogon citratus*	Leaf and root	Nigeria	*P. berghei*	Aqueous	Infected mice	Chloroquine	The aqueous leaf extracts have suppressive effect of 20.83%, 55.56%, and 80.56%, root extracts have 50.38%, 77.78%, and 100%	[67]
15	*Carica papaya* and *Vernonia amygdalina*	Leaf extracts	Nigeria	Chloroquine-sensitive *P. berghei* (Nk65)	Aqueous	Infected mice	Halofantrine	Significant (*P* < 0.05) reduction in the percentage of parasite load between the infected treatment groups and disease control group at day 3	[68]
16	*Mangifera indica, Psidium guajava, Carica papaya, Cymbopogon citratus, Citrus sinensis,* and *Ocimum gratissimum*	Bark and leaf	Cameroon	*P. falciparum*	Aqueous and ethanol	3% hematocrit in human red blood cells	Chloroquine and artemisinin	The derived EC_50_ (3D7/Dd2, g/mL) are nefang 96.96/55.08, MiB-65.33/34.58, MiL-82.56/40.04, Pg-47.02/25.79, Cp-1188/317.5, Cc-723.3/141s and og-778.5/118.9	[69]
17	*Aloe megalacantha*	Leaf	Ethiopia	*P. berghei*		Swiss albino mice	Chloroquine	Parasite suppression of day 1 (30.3%, 43.4%, and 56.4%), day 2 (32.3%, 51.3%, and 67.4%), day 3 (39.8%, 50.6%, and 64.2%), day 4 (52.6%, 69.4%, and 79.6%) was observed at doses of 100, 200, and 400 mg/kg/day	[70]
18	*Aloe vera*	Leaf	India	*P. falciparum* (MRC-2).	Aqueous		Chloroquine	The EC_50_ of 0.289 to 1056 *μ*g/ml. The antiplasmodial EC_50_ of chloroquine was 0.034 *μ*g/ml and aloin and aloe-emodin was 67 *μ*g/ml and 22 *μ*g/ml, respectively	[11]
19	*Carica papaya*	Fruit rind and root	Ethiopia	*P. berghei*	Pet ether, chloroform, and methanol	Male Swiss albino mice	Chloroquine	Suppression of 61.78% was produced by pet ether fraction of *C. papaya* fruit rind, chloroform fraction of *C. papaya* root exhibited (48.11%), methanol fraction produced less effect	[71]
20	*Mangifera Indica*	Leaf	Nigeria	*P. Berghei*	Aqueous	Infected albino mice	Artesunate	The extract has a dose-dependent reducing effect on the level of parasitaemia	[72]
21	*Stemonocoleus micranthus*	Stem bark	Nigeria	*P. berghei*	Hydromethanol	Swiss albino mice	Chloroquine (positive)	Chemosuppressive effect ranged from 54.14 to 67.73% and 59.41 to 94.51%	[73]
22	*Lawsonia inermis, Tithonia diversifolia,* and *Chromolaena odorata*	Leaf	Nigeria	*P. berghei* ANKA	Dichloromethane, methanol	Swiss albino mice	Chloroquine and artemisinin	IC_50_ of 0.437 ± 0.02 mg/mL and 2.557 ± 0.19 mg/mL against D6 and W2, respectively	[43]
23	*Holarrhena antidysenterica* and *Azadirachta indica*	Leaves, stem, bark	India	*P. berghei*	Aqueous	Mycoplasma free male Swiss mice	Chloroquine	The parasitaemia increased gradually in all the groups, with the maximum in the control group (day 3–35, day 9–46.98) and minimum in chloroquine arm (day 3–14.06, day 9–19.92)	[41]
24	*Euphorbia hirta* and *Vernonia amygdalina*	Whole plant, leaves	Nigeria	*P. berghei*	Ethanol	Infected mice	Camosunate, ACT	ACT was slightly potent (>50%) against chloroquine-sensitive *P. berghei*	[74]
25	*Pseudocedrala kotschyi*	Leaf	Nigeria	*P. berghei*	Ethanol	Swiss Albino mice	Chloroquine	The leaf extract exhibited significant dose-dependent activity against the parasite in the suppressive and curative activity	[75]

**Table 5:** The isolated compounds from medicinal plants used as antimalarial.

S/n	Name of plant	Phytochemical compounds	Structure	Reference
1	*Morinda lucida*	Asperulosidic acid	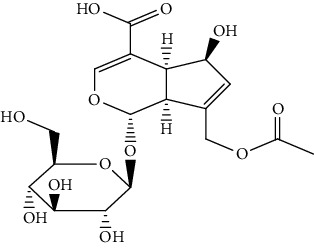	[46]

2	*C. citratus*	Geranial	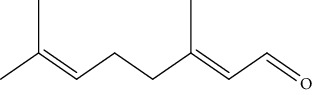	[55]

3	*Aloe vera*	6′-Malonylnataloin (nataloin)	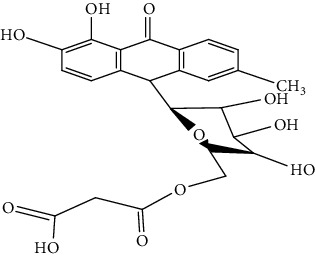	[79]

4	*Fagara zanthoxyloides*	Fagaronine	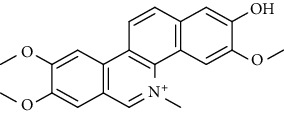	[84]

5	*Enantia chlorantha*	Jatrorrhizine	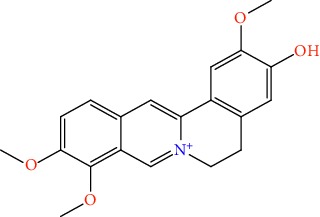	[85]

6	*Azadirachta indica*	Gedunin	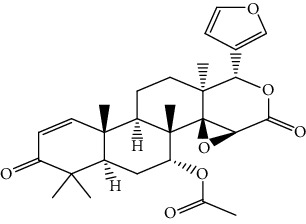	[86]

7	*Morinda lucida*	Asperuloside	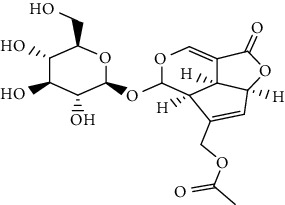	[46]

8	*Aloe vera*	7-Hydroxyaloin B	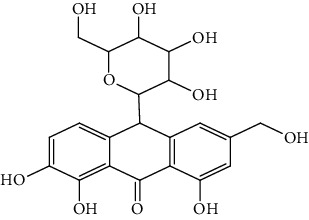	[79, 80]

9	*Khaya grandifoliola*	Methyl angolensate	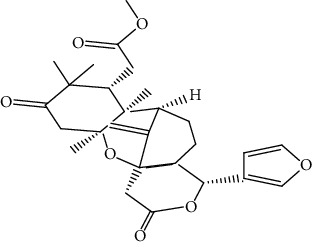	[87]

10	*Khaya senegalensis*	Fissinolide	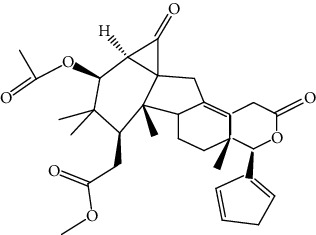	[88]

11	*Azadirachta indica*	Meldenin	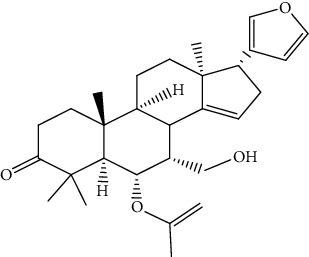	[89]

12	*Morinda lucida*	Campesterol	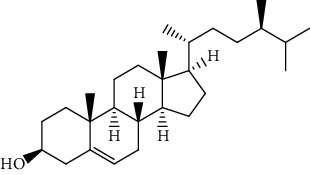	[46]

13	*Quassia amara*	Simalikalactone D	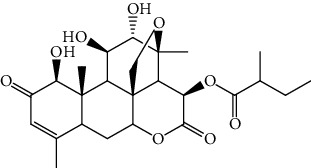	[90]

14	*Picralima nitida*	Akuammiline	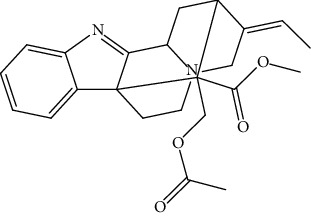	[91]

15	*Morinda lucida*	Cycloartenol	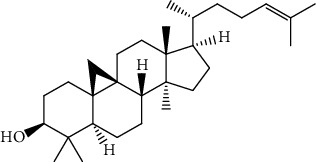	[46]

16	*Jatropha multifida*	Multifidinol	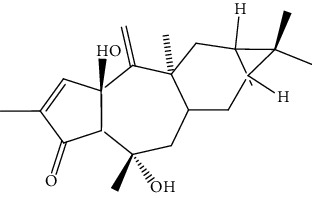	[41]

17	*E. chlorantia*	Ergosterol	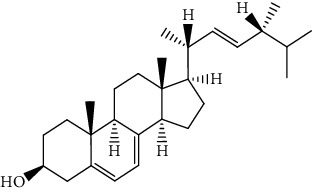	[36]

18	*Cylicodiscus gabunensis*	3,4,5-Trihydroxybenzoic acid	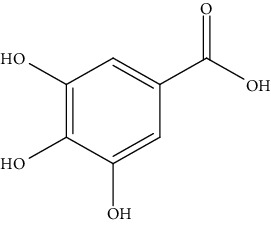	[92]

19	*Morinda lucida*	Stigmasterol	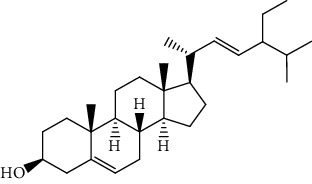	[46]

20	*Picralima nitida,*	Akuammigine	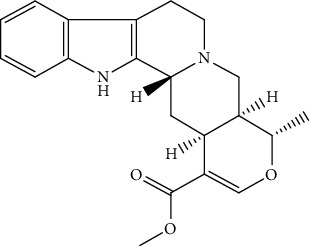	[91]

21	*Diospyros conocarpa*	Mangiferolic acid	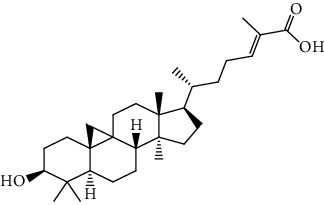	[93]

22	*Antrocaryon klaineanum*	Antrocarine A	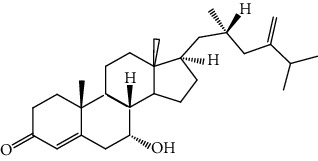	[93]

23	*C. papaya*	Anacardic acid	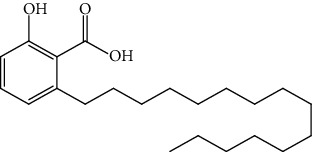	[94]

24	*Picralima nitida,*	Alstonine	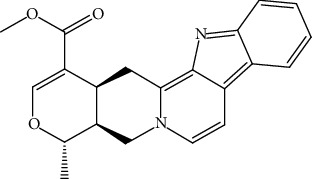	[91]

25	*C. papaya*	Cardol triene	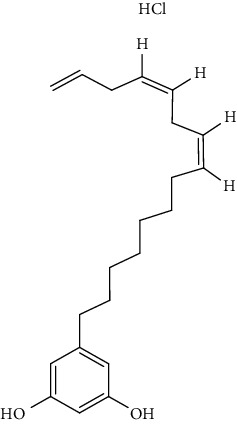	[94]

## Data Availability

The datasets used and/or analysed during the current study are available in the manuscript and others not included are available from the corresponding author upon request.
